# Rapamycin for longevity: opinion article

**DOI:** 10.18632/aging.102355

**Published:** 2019-10-04

**Authors:** Mikhail V. Blagosklonny

**Affiliations:** 1 Cell Stress Biology, Roswell Park Cancer Institute, Buffalo, NY 14263, USA

**Keywords:** rapamycin, rapalogs, metformin, aging, anti-aging, fasting, lifespan, health span

## Abstract

From the dawn of civilization, humanity has dreamed of immortality. So why didn’t the discovery of the anti-aging properties of mTOR inhibitors change the world forever? I will discuss several reasons, including fear of the actual and fictional side effects of rapamycin, everolimus and other clinically-approved drugs, arguing that no real side effects preclude their use as anti-aging drugs today. Furthermore, the alternative to the reversible (and avoidable) side effects of rapamycin/everolimus are the irreversible (and inevitable) effects of aging: cancer, stroke, infarction, blindness and premature death. I will also discuss why it is more dangerous not to use anti-aging drugs than to use them and how rapamycin-based drug combinations have already been implemented for potential life extension in humans. If you read this article from the very beginning to its end, you may realize that the time is now.

**“If you wait until you are ready, it is almost certainly too late.”** Seth Godin

In one short-lived mutant strain of mice, the mTOR inhibitor rapamycin (known in the clinic as Sirolimus) extends maximum life span nearly three-fold [1]. Albeit less spectacularly, rapamycin also prolongs life in normal mice as well as in yeast, worms and flies, and it prevents age-related conditions in rodents, dogs, nonhuman primates and humans. Rapamycin and its analog, everolimus, are FDA approved for human use and have been used safely for decades. In 2006, it was suggested that rapamycin could be used immediately to slow down aging and all age-related diseases in humans [2], becoming an “anti-aging drug today” [3].

## But rapamycin was unlucky

Rapamycin known in the clinic as Rapamune or Sirolimus, was unlucky from the start, however. Twenty years ago, it was labeled an immunosuppressant and used to treat renal transplant patients. If rapamycin had been labeled an immunomodulator and anti-inflammatory drug instead, it would sound much more appealing now. At anti-aging doses, rapamycin “eliminates hyperimmunity rather than suppresses immunity” or, more figuratively, it “rejuvenates immunity” [2]. This enables rapamycin and everolimus, a rapamycin analog, to act as immunostimulators [4–6], improving immunity in cancer patients [7] and the elderly [8,9]. For example, rapamycin reduces the risk of CMV infection in organ transplant patients [10–12], improves antipathogen and anticancer immunity in mice [13–15], prolongs lifespan in infection-prone mice [16] and protects aged mice against pneumonia [17]. Rapamycin also inhibits viral replication [18,19]. As a noteworthy example, rapamycin inhibits replication of the 1918 flu virus (the deadliest flu virus in history) by 100-fold [19], and also protects against lethal infection with influenza virus when administered during vaccination [13]. Still, as Dr. Allan Green advises, patients taking rapamycin should be carefully monitored for skin and subcutaneous bacterial infections, which should be treated with antibiotics https://rapamycintherapy.com.

Twenty years ago, it was thought that rapamycin might increase the risk of cancer (see a forthcoming review “Understanding the side effects of rapamycin”). Despite that concern, it was revealed that rapamycin actually prevents lymphoma and some types of cancer in transplant patients [20–27]. Currently, in fact, rapamycin analogs, everolimus and temsirolimus, are widely used in cancer therapy. Furthermore, rapamycin is the most effective known cancer-preventive agent in mice [25,28–32] extending the lifespan of cancer-prone mice [33–36]. It has even been suggested that rapamycin extends lifespan by preventing cancer [37].

Nevertheless, social media often warn that although rapamycin prevents cancer, its use to prevent cancer may come at the cost of getting cancer. This self-contradiction miscites a twenty-year-old warning by the FDA for all drugs marketed as immunosuppressants (including rapamycin and everolimus): “Increased susceptibility to infection and the possible development of malignancies such as lymphoma and skin cancer may result from immunosuppression.” This statement does not say that rapamycin or everolimus cause malignancies. (Just read it again). Although rapamycin and its analogs are now approved by the FDA for treatment of cancer and lymphomas, the rumors that these drugs may cause cancer persist. To my knowledge, no study has shown that mTOR inhibitors cause cancer.

At this point, most scientists agree that rapamycin is not counterindicated because of concerns about immunosuppressive effects. But a new objection against rapamycin has emerged, namely that rapamycin may cause diabetes. As discussed in detail [38], the new wave of “fear of rapamycin” is groundless. So, what are the metabolic effects of rapamycin?

## Metabolic effects or rapamycin and starvation

When it is over-activated by nutrients and insulin, mTOR acts via S6K to inhibit insulin signaling, thereby causing insulin resistance [39–44]. Acute treatment with rapamycin abrogates insulin resistance in cells and animals including humans [45–51]. One study showed that chronic treatment with rapamycin may also prevent insulin resistance [52]. However, in some (but not all) rodent models, chronic treatment with rapamycin can also cause glucose intolerance and even insulin resistance [53–56]. This was interpreted as a deleterious side effect or even type 2 diabetes (T2D). Actually, however, these metabolic changes are features of benevolent starvation pseudo-diabetes (SPD), which was described 170 years ago in fasted animals and later in humans [57,58]. During prolonged fasting, utilization of glucose by non-brain tissues must be suppressed to ensure an adequate supply to the brain. When a fasted animal or human is then given a meal, glucose appears in the urine (glycosuria), which is a canonical symptom of diabetes. But this is because prolonged fasting (starvation) leads to decreased insulin secretion and to insulin resistance, and subsequent re-feeding then causes transient hyperglycemia and glycosuria. This SPD can be caused not only by prolonged fasting, but also by severe restriction of calorie and carbohydrate intake [38]. For example, severe calorie restriction can cause diabetes-like glucose intolerance [59]. Despite that, very low calorie diets are the most effective treatments for type 2 diabetes [60–62]. Some researchers re-discovered SPD in obese patients on strenuous weight loss program and erroneously warned that low calorie diets cause type 2 diabetes [63].

The obligatory symptom of starvation is ketosis, as ketones substitute for glucose as the main fuel for the brain. The ketogenic diet, a promising treatment for diabetes and obesity in humans, can cause symptoms of SPD in rodents (see for references [64]). Once again, some researchers warned that the ketogenic diet can favor type 2 diabetes [65]. As discussed, such warnings may not be justified [64,66–68].

Rapamycin can be viewed as a partial starvation-mimetic [69–71]. It is therefore predictable that, under some conditions, prolonged treatment with rapamycin may lead to the emergence of SPD [72]. This has been confirmed in rapamycin-fed mice, which developed insulin resistance, glucose intolerance and mild hyperglycemia [54]. Nevertheless, rapamycin-fed mice lived longer and thus were healthier than mice fed a standard diet [54]. It is not completely clear why SPD was observed in only some studies and was not observed in other studies (see for references [38,73]).

Importantly, SPD is reversible and does not lead to complications. Furthermore, rapamycin reduces the incidence of diabetic complications such as diabetic nephropathy in rodents [42,74–85]. In healthy elderly humans, chronic treatment with rapamycin or everolimus did not cause hyperglycemia [8,9,86]. On the contrary, the risk of hyperglycemia was lower in the mTOR inhibitor treatment group than the placebo group [9].

In some cancer patients, high doses of rapamycin or everolimus can cause hyperglycemia, which is usually mild (grade 1-2) and reversible, and does not lead to treatment interruption [87–89]. Hyperglycemia is a common side effect of many oncotargeted drugs and is not a manifestation of diabetes. Everolumus, for example, can cause hyperglycemia by decreasing insulin production [89].

To summarize, chronic treatment with high doses of rapamycin may cause symptoms of reversible SPD. Diet-induced SPD, at least, is beneficial and therapeutic. Rapamycin-induced SPD is a relatively rare side effect and probably can be avoided by administering the drug intermittently or at lower doses, and if SPD does occur, it can be reversed by discontinuation of the drug.

In some studies in transplant patients, rapamycin (sirolimus) and everolimus did not increase the risk of diabetes [90–96]. In one study, no patient, out of 21 patients treated with rapamycin, developed diabetes, while the incidence of diabetes was 7% in patients treated with either cyclosporine or tacrolimus [96]. Most importantly, cyclosporine- or tacrolimus-induced diabetes resolved in 80% of patients after conversion from tacrolimus/cyclosporine to rapamycin (sirolimus) [96].

On the other hand, a large retrospective study reported an association between Medicare billing for diabetes treatment and rapamycin use, implying that rapamycin may increase the risk of diabetes [97]. However, this association was explained by the interaction between rapamycin and calcineurin inhibitors, which increase each other’s levels [96,98,99]. Consequently, it remains unclear whether rapamycin per se increases the risk of diabetes in transplant patients [96]. Moreover, this is further complicated by the fact that most transplant patients develop type 2 diabetes spontaneously without rapamycin treatment [100].

## Rapamycin is not much more dangerous than ordinary drugs

**Figure 1:**
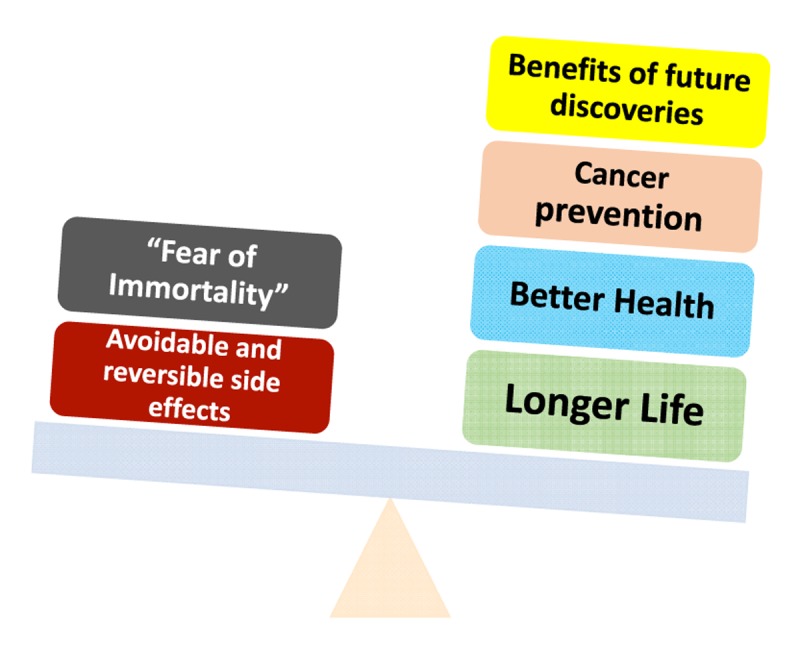
**Potential risk vs benefits of rapamycin-based anti-aging therapy.** Pros and Cons: Potential benefits of rapamycin may outweigh its risks.

If used properly, rapamycin is not much more dangerous than ordinary aspirin. Aspirin, one of the most widely used nonprescription medications, may cause numerous side effects, including life threatening gastric bleeding. The manufacturer lists as possible side effects: ringing in ears, confusion, hallucinations, seizure, severe nausea, vomiting, bloody stools, coughing up blood, fever and swelling. Still, millions of people take aspirin daily to prevent cardiovascular disease and cancer. It was calculated that the benefits of aspirin are greater than their risks [101,102]. I believe the benefits of the anti-aging effects of rapamycin/everolimus may even be greater (Figure 1).

In the case of rapamycin and everolimus, the most worrying side effects have not been confirmed. At low doses [8,9,86], or when administered as a single high dose [103], no side effects have been detected so far in the elderly. At high doses, rapamycin and everolimus slow cell proliferation, which decreases blood cell counts. As a result, mild and reversible thrombocytopenia (low platelet count), anemia and leukopenia are their most common side effects. But a mild reduction of platelets may be beneficial. In fact, one of the intended effects of aspirin is a decrease in platelet function.

There is one crucial reason why the side effects of rapamycin are exaggerated. The frequency of rapamycin side effects has often been estimated in studies lacking a placebo group. In cancer and transplant patients, numerous effects ascribed to rapamycin, such as fatigue (asthenia), for example, are often caused by the disease itself. In a placebo study of healthy volunteers, the placebo group reported more side effects such as fatigue than did the rapamycin group [104]. In recent placebo-controlled studies in healthy elderly people, no side effects were noticed as compared to placebo [9,86].

While aspirin may cause gastric ulceration and bleeding, rapamycin may cause stomatitis and mycositis (ulceration of the mucous membranes of the mouth and the digestive tract) when used at high doses or chronically. A rare side effect of rapamycin is noninfectious interstitial pneumonitis [105]. And by inhibiting neutrophil function, rapamycin may increase the severity of bacterial infections [106]. These side effects require rapamycin’s discontinuation. For antiaging purposes, however, rapamycin may be used either intermittently (e.g., once a week) or at low daily doses and can be discontinued if any unpleasant effects occur.

## From a single dose to intermittent schedules

Although nearly all drugs, including nonprescription drugs such as aspirin, can be fatal at sufficiently high doses, there are no known fatal cases of acute rapamycin (sirolimus) overdose [103]. For example, in a failed suicide attempt, an 18-year-old woman ingested 103 rapamycin tablets (103 mg), and the only detected effect was an elevation in total blood cholesterol [103]. In rats, rapamycin’s LD50, a measure of drug lethality, could not be determined because it is higher than 2500 mg/kg. While a single dose of rapamycin is safe, it is sufficient to extend life and decrease obesity in several rodent models [1,107]. Furthermore, transient treatment with rapamycin can be long lasting, extending the lifespan and preventing obesity long after drug discontinuation [107–112]. The magnitude of life extension by rapamycin depends mostly on reaching a high peak blood level [113]. A similar conclusion was reached by a study of rapamycin use in obesity [112]. It was suggested in 2008 that a pulse (intermittent) schedule of rapamycin administration would improve regeneration of stem cells [114] while avoiding mTORC2 inhibition [54,115].

**Figure 2:**
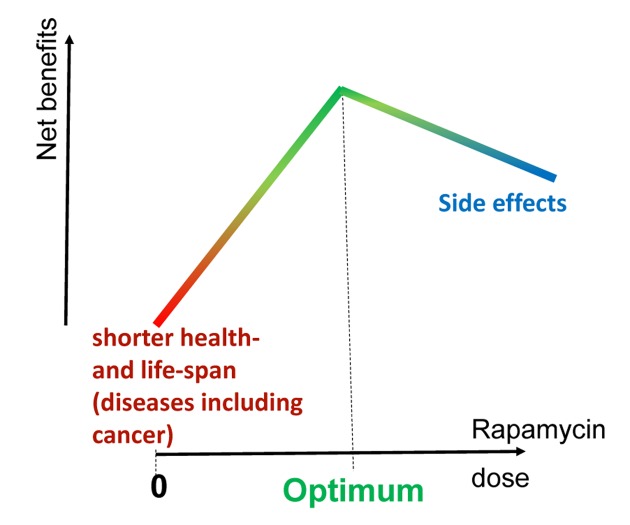
**Optimal dose of rapamycin for maximal net benefits.** Life extension by rapamycin is dose-dependent in rodents. The higher the dose, the higher the anti-aging benefits, including cancer prevention and life extension. In humans, side effects are dose-dependent and net benefits could potentially decrease at very high doses. This point of the highest net benefit is the optimal dose. The optimal dose varies in different individuals due to the variability of potential side effects. Thus, the optimal dose in a particular individual is determined by the emergence of side effects. The treatment can be viewed as life-long phase I/II clinical trial.

Therefore, to avoid side effects and maximize anti-aging effects [110], a feasible approach would be to prolong intervals between rapamycin administrations while keeping the total dose constant. For example, instead of daily administration, a weekly administration of a higher dose can be suggested to achieve a high peak blood level, followed by drug-free period to avoid undesirable effects. Still, everyday treatment of the elderly (1 mg/day for several weeks) was not associated with side effects and has been shown to be safe [86]. Similar results were achieved with low doses of other mTOR inhibitors [9]. Another option is an alternating schedule; for example, a 3- month course of weekly rapamycin alternating with a rapamycin-free month. Finally, anti-aging schedules can be very flexible to fit an individual patient. The optimal anti-aging dose is a personalized maximum dose that does not cause side effects in a particular patient (Figure 2).

In conclusion, the side effects of rapamycin are well-known and reversible. When used on an anti-aging schedule, side effects may be absent but, if not, they may be mitigated by combining rapamycin with other anti-aging drugs (metformin, statins) or by temporarily discontinuing it.

Noteworthy, the alternative to the reversible (and avoidable) side effects of rapamycin/everolimus are the irreversible (and inevitable) effects of aging. And by living longer, our generation will benefit from future anti-aging discoveries (Figure 1).

But the fear of nonexistent side effects is not the only reason the use of mTOR inhibitors for life extension has been questioned. The second reason is that there is rightful skepticism about any claims made about anti-aging drugs because thousands of anti-aging remedies have already failed. What then makes rapamycin different?

## The history of mankind: empty promises of immortality

On the one hand, from the dawn of civilization humans have dreamed of immortality. On the other hand, from the dawn of civilization a myriad of anti-aging remedies turned out to be empty promises. Even worse, they often shorten lifespan. Two notable examples are antioxidants and human growth hormone. The idea that free radicals, or reactive oxygen species (ROS), cause aging was based on a “wild guess,” as Harman, a father of the ROS theory, acknowledged when he titled his paper, “I thought, thought, thought for four months in vain and suddenly the idea came” [116]. The idea is simple and intuitive, and it was widely accepted based on circumstantial evidence. In fact, ROS are inevitable products of metabolism, and they do damage biomolecules. Moreover, *excessive* ROS can shorten lifespan. Similarly, the atomic bomb can shorten life span. Yet this does not mean that either atomic bombs or oxidants are the cause of normal aging as we know it.

Numerous experiments support the ROS theory. However, key experiments ruled the ROS theory out (see for references [2,117–122]. To make a long story short, antioxidants could in theory prolong lifespan if mTOR-driven (quasi-programmed) aging were suppressed and we lived long enough to die from ROS-induced post-aging syndrome (I will discuss the nuances in the forthcoming article “ROS and aging revisited”). Indeed, ROS will kill any organism eventually. However, organisms normally die from mTOR-driven, age-related diseases (aging as we know it) before ROS can kill them (see for discussion [2]). As an analogy, consider most of the passengers on the Titanic. Would antioxidant treatment have been useful to them for life extension? The best way to extend life for members of that group would have been to carry more life boats. Only after their safe rescue could one expect antioxidants to potentially increase their life further. Similarly, only after rescue from the quasi-program of aging may antioxidants potentially have an impact.

Not surprisingly, antioxidants did not extend lifespan in any clinical trials and were detrimental in some [122–133]. As Ristow put it, they were “worse than useless” [119]. For example, in two very large randomized controlled trials, antioxidants increased the incidence of cancer, especially of lung cancer in smokers [131–133]. Antioxidants also increased all-cause mortality. The results were so disturbing that two trials were stopped earlier than planned [131–133]. Also disturbing is the finding that antioxidants accelerate cancer progression and promote metastasis [134–136]. But despite their uselessness, antioxidants continue to be a multibillion-dollar business. They are widely sold as natural products in the forms of nutritional supplements and in foods “rich in antioxidants.”

Another example is human growth hormone (HGH), which is widely used for rejuvenation and longevity. Yet, it actually accelerates aging and shortens lifespan [137,138]. Growth hormone is a pro-aging hormone because it indirectly activates mTOR [139]. Notably, the hype around growth hormone is based on a single publication [140], which misinterpreted its acute effects [141].

Given that all previous anti-aging remedies have failed to meet expectations, it is not surprising that the discovery of the anti-aging effects of rapamycin are being met with skepticism too. But unlike HGH, the effects of rapamycin are not based on one single paper as were HGH, nor is it based on a wild guess as were ROS.

## Rapamycin is a proven anti-aging drug

The evidence that rapamycin can function as an anti-aging drug is the product of thousands of scientists working independently all over the world, studying mTOR and its inhibitors for a variety of different reasons in diverse organisms, ranging from yeast to humans. Studies in model organisms, such as yeast, worms and flies, have revealed components of the TOR signaling pathway [142–145]. It was predicted in 2003 [146] that conversion from quiescence to senescence (geroconversion) is driven by growth-promoting mediators, such as mTOR, when the cell cycle is blocked [147]. Figuratively, geroconversion is “twisted” growth that occurs when actual growth is completed [2], [147]. In cell culture, mTOR is maximally activated and geroconversion lasts 3-6 days, whereas in the human body it may take decades. mTOR drives geroconversion, rendering cells hypertrophic and hyperfunctional (e.g. senescence-associated secretory phenotype), which eventually leads to the development of age-related pathologies [2]. Working independently, clinical researchers have studied rapamycin for the prevention and treatment of nearly every age-related disease, including cancer, obesity, atherosclerosis and neurodegeneration. If a drug is indicated for all age-related diseases, it must be an anti-aging drug in that it targets a common driver of age-related diseases – that is, aging (see for references [2]). This is because aging is the sum of all age-related diseases, which limit lifespan [148–150]. Does rapamycin suppress aging and extend lifespan by preventing diseases, or does it prevent diseases by slowing aging? Actually, both reflect the same process.

By 2006, an extensive body of work from several independent fields all pointed to rapamycin as an anti-aging drug [2]. According to hyperfunction theory, aging is an unintended (not programmed but quasi-programmed) continuation of the developmental growth program, driven in part by mTOR [2,120,121,151,152]. Testable predictions have been formulated [2,153] and confirmed in numerous independent studies (see for references: [150,154]).

In two dozen studies using different strains of mice, rapamycin extended life span. Starting from a thorough study by Harrison et al. [155] and followed by nearly simultaneous studies by others [33,108], the anti-aging effects of rapamycin have been confirmed many times (see for references: [113,150,156,157]). Importantly, rapamycin and everolimus are indicated in most, if not all, age-related diseases, from cancer to neurodegeneration [2,158].

## Conventional drugs as anti-aging agents

Several conventional drugs used to treat age-related diseases (e.g., hypertension, ischemic heart disease, diabetes, cancer, prostate enlargement) can be viewed as somewhat anti-aging drugs [150,154]. First, these drugs extend lifespan in the same model organisms (see for references: [159]). For example, metformin extends lifespan not only in mice, but also in the worms, which do not suffer from human diseases [160,161]. ACE inhibitors prolong life not only in hypertensive rats, but also in healthy normotensive rats [162]. If these drugs were not ordinary drugs for human diseases, then gerontologists would call them anti-aging agents.

Second, these drugs prevent or treat more than one disease. For example, metformin is indicated to treat type 2 diabetes as well as pre-diabetes, obesity, metabolic syndrome, cancer, and polycystic ovary syndrome [163–168]. Aspirin not only reduces inflammation (a hallmark of aging), it also reduces the risk of cardiovascular disease, thrombosis and cancer. Low-dose aspirin prevents one-third of colorectal, gastric, and esophageal cancers [169]. PDE5 inhibitors such as Sildenafil and Tadalafil, which are widely used for erectile dysfunction, are also effective against benign prostatic hyperplasia (BPH) and pulmonary arterial hypertension in humans and suppress inflammation-driven colorectal cancer in mice [170]. Aging is the sum of all these age-related diseases. Given that humans and animals die from age-related diseases, life can be extended by treating multiple pre-diseases and diseases. Rapamycin and these drugs may complement each other in an anti-aging formulation by further extending life and/or by mitigating each others possible side effects [159]. For example, metformin may counteract rapamycin-induced hyperglycemia [171].

## Not taking rapamycin may be as dangerous as smoking

Strangely, the fear of tobacco smoking is less intense than the fear of rapamycin. But whereas smoking shortens both the healthspan and lifespan, rapamycin extends them. Smoking increases the incidence of cancer and other age-related diseases. Rapamycin prevents cancer in mice and humans. Heavy smoking shortens life expectancy by 6-10 years. In other words, simply *not* smoking prolongs life by 6-10 years. In middle-aged mice, just 3 months of high-dose rapamycin treatment was sufficient to increase life expectancy up to 60% [109]. When taken late in life, rapamycin increases lifespan by 9-14% [155], despite the dosage being suboptimal [111]. This possibly equates to more than 7 years of human life. By comparison, smokers who quit late in life (at age 65 years), gain between 1.4 -3.7 years [172]. Considered in those terms, one could say that in the elderly, *not* taking rapamycin may be even more “dangerous” than smoking. Finally, rapamycin may be especially beneficial to smokers and former smokers. While the carcinogens from tobacco cause lung cancer in mice, rapamycin decreases tobacco-induced lung cancer multiplicity by 90% [28].

## Diet and rapamycin

Calorie restriction (CR) and intermittent fasting (IF) extend both the lifespan and healthspan in diverse species. However, CR is of little benefit when started in old age [73,173–178]. Fasting inhibits the mTOR pathway in young but not old mice [179,180]. By contrast, rapamycin strongly inhibits mTORC1 at any age. It extends lifespan, whether started late or early in life [108,155,181], even if used transiently [109]. So, whereas CR is more beneficial early in life, rapamycin may be indicated later in life. In addition, the beneficial effects of rapamycin and CR may be additive, given that they are exerted through overlapping but distinct mechanisms [182–186]. Intermittent rapamycin and CR (24-48 hours after) can be combined, to avoid potential hyperglycemia. Physical exercise may be most beneficial starting immediately after rapamycin use, to take advantage of rapamycin-induced lipolysis as a fuel for the muscles. By itself, chronic rapamycin treatment does not compromise muscle endurance [187] and even prevents muscle loss [188–190].

## Do we need new or safer rapalogs to start aging prevention?

Despite the metabolic side effects seen in some mouse models, mice treated with rapamycin live longer and are healthier. Humans also may want to live longer and healthier lives, regardless of whether one calls the means unsafe. Some basic researchers believe that rapamycin cannot be routinely used to treat aging in humans because of its metabolic effects and call for the development of safer analogs. First, rapamycin and everolimus are FDA-approved drugs, safe for human use. Since 1999, rapamycin has been used by millions of patients with no unexpected problems. One may suggest that rapamycin/everolimus are safe enough for very sick patients, not for healthy people.

First, healthy elderly people chronically treated with rapamycin or other mTOR inhibitors showed no ill effects (e.g. hyperglycemia) [8,9,86]. Logically, more threatening adverse effects could be expected in cancer and transplant patients, who are often heavily pre-treated and terminally ill than in healthy people. Second, there are no truly healthy people among the elderly; otherwise, they would be “immortal”, given that all humans die from age-related diseases, not from healthy aging. And the sooner they would be treated with anti-aging drugs, the longer they would remain relatively healthy.

That said, it is, of course, important to develop new rapalogs, but not because current rapalogs are unsafe. It is important because such research will help us to learn more about mTOR and aging and may lead to the discovery of agents capable inhibiting the rapamycin-insensitive functions of mTORC1. These future drugs could potentially complement current rapalogs to further extend lifespan. Non-rapalog rapamycin analogs will also be developed [191]. The limitation of current rapalogs is not that they are unsafe but that their ability to extend life is limited. The goal should be to develop new drugs that extend life span further.

Rapamycin is a natural anti-fungal antibiotic produced by soil bacteria of Eastern Island. The patent on rapamycin has expired, and pharmacological companies have developed other rapalogs such as everolimus. (I use the term rapalogs to encompass both rapamycin, everolimus and any other analogs). At equipotent doses, rapamycin and everolimus exert almost identical therapeutic and adverse effects; although, everolimus is weaker and has a shorter half-life in the organism compared with rapamycin.

All current rapalogs exhibit the same side effects as rapamycin and everolimus. Their real side effects are mTORC1-dependent. Inhibition of mTORC1 decreases cell proliferation and function, which is manifested as lower blood cell counts and insulin levels, especially when rapalogs are chronically administered at high doses. We could develop weaker rapalogs, which would have no side effects if used at the same dose as rapamycin. But then why not just use a lower dose of rapamycin? (I will discuss elsewhere how safer rapalogs are probably weaker rapalogs.) Given to mice at the same doses as rapamycin, weaker analogs would have neither side effects and no therapeutic effects. Consequently, their metabolic effects would be diminished and so would their therapeutic effects. However, the same negative result can be achieved simply by decreasing the dose of rapamycin. While waiting for silver bullets, we need to use the currently available rapalogs, such as rapamycin and everolimus, to live longer. When “safer” rapalogs are clinically available, we may use them too.

## The time is now unless it’s too late

**Figure 3:**
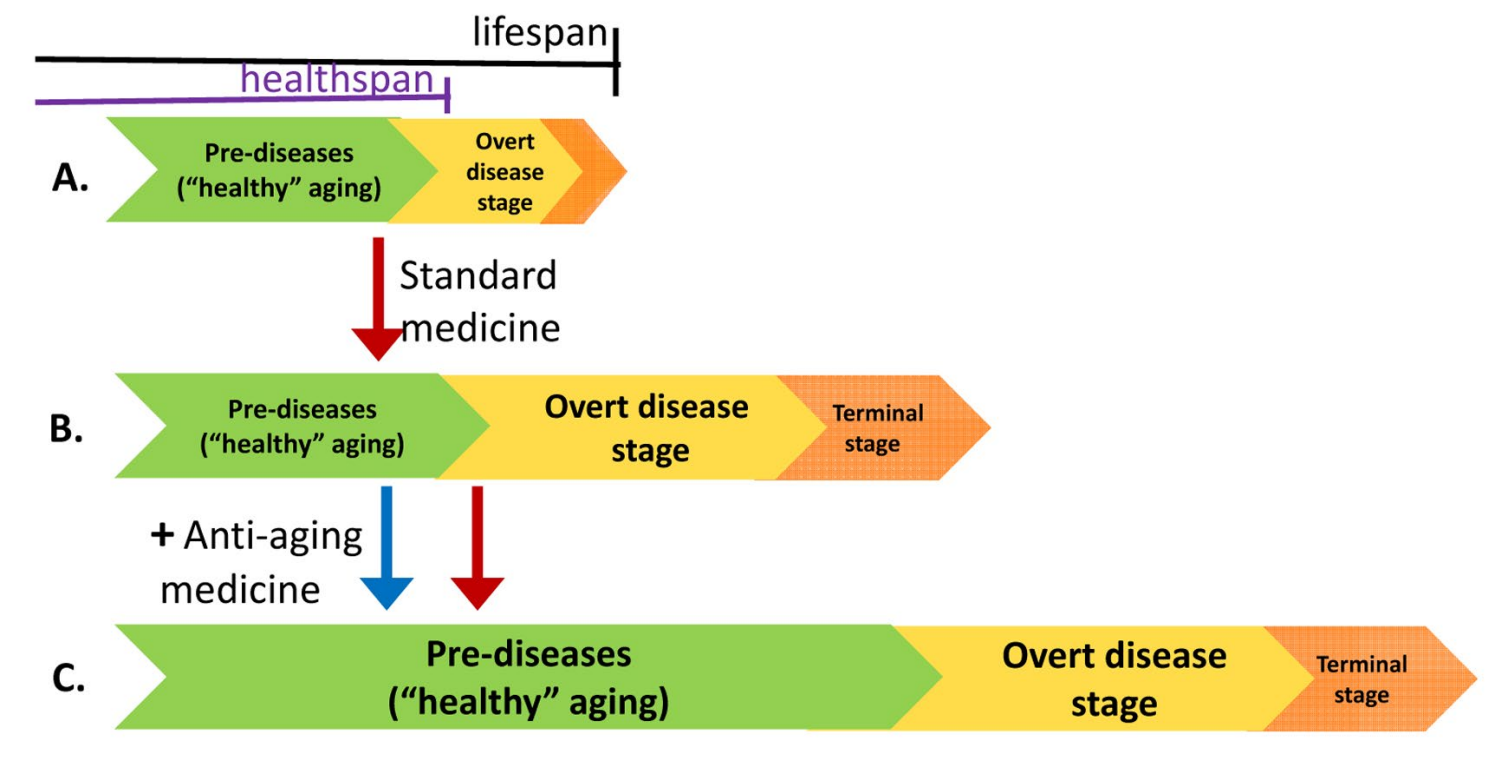
**Effects of standard and anti-aging medicine on health- and life-span.** (**A**) The relationship between health- and life-span. Aging is a sum of all age-related diseases, pre-diseases and pre-pre-diseases. Before overt age-related diseases become apparent, there is a seemingly healthy period of aging (so-called healthy aging). Starting from adulthood, pre-pre-diseases progress towards pre-diseases and then towards overt diseases. Unless treated with modern standard medical practice, the diseased stage is relatively brief. From (**A**) to (**B**) Standard medical treatment is usually started when overt diseases are diagnosed. Standard medicine extends life span mostly by preventing death from diseases, thus extending “unhealthy” phase of life, especially terminal stages of diseases, characterized by organ damage, failure and loss of functions. Standard medicine extends lifespan. From (**B**) to (**C**) Anti-aging medicine is most effective at the stage of pre-diseases and initial stages of diseases, characterized by increased functions before complications and organ damage occur. In terminal stages of deadly diseases, anti-aging therapy may not be useful. Thus, anti-aging medicine increases both health span and life span. Anti-aging medicine and standard medicine are additive when aging becomes unhealthy. The schema is simplified because, in reality, age-related diseases start at different ages (presbyopia vs sarcopenia), progress at different paces (atherosclerosis vs cancer), and most are not lethal, and some are well treated (cataract). Therefore, healthspan is an abstraction.

The overwhelming evidence suggests that rapamycin is a universal anti-aging drug – that is, it extends lifespan in all tested models from yeast to mammals, suppresses cell senescence and delays the onset of age-related diseases, which are manifestations of aging [discussed by me in [148,149,158,192]. Although rapamycin may reverse some manifestations of aging [181,193], it is more effective at slowing down aging than reversing it. Therefore, rapamycin will be most effective when administered at the pre-disease, or even pre-pre-disease stages of age-related diseases [150]. For example, Carosi et al. suggested that mTOR inhibitors could be useful in Alzheimer disease, but only in the earliest stages [194,195]. In addition, rapamycin and everolimus are more effective for preventing cancer than treating it. They may also be useful for treating osteoporosis, though not a broken hip after an osteoporotic fracture. Rapalogs may slow atherosclerosis, thereby preventing myocardial infarction, but they are unlikely to help reverse an infarction. In other words, anti-aging drugs extend the healthspan (Figure 3) and are most effective before overt diseases cause organ damage and loss of function.

So, is it too late to take rapamycin once aging reaches an unhealthy stage? Actually, it is not too late. Even if one or a few age-related diseases renders aging unhealthy, other potential diseases are still at pre-disease stages, and anti-aging drugs may delay their development. And they may slow down further progression of existing overt diseases.

In addition to rapamycin/everolimus, the anti-aging formula metformin, aspirin, ACE inhibitors, angiotensin receptor blockers and PDE5 inhibitors, each of which can prevent or treat more than one age-related disease [159]. Note that I mention only clinically-approved drugs because they can be used now. Later, perhaps, we may be able to consider further life extension through the use of low doses of pan-mTOR [196,197], mdm-2 [198,199] and MEK inhibitors [200,201], lithium [201,202], as well as next-generation rapalogs.

There is currently no consensus around the short-term markers of anti-aging effects. Therefore, rapamycin trials should be focused on its potential side effects rather than anti-aging effects. We must be sure that the therapy is safe. In the future, the treatment should be conducted as a life-long phase I/II trial, with dose escalation of rapamycin/everolimus until the side effects are reached in an individual patient. The tailored optimal dose (see Figure 2) should be determined individually for each patient and may vary widely. Doses and frequencies should be limited by the side effects: stomatitis/mucositis, anemia, thrombopenia, leukopenia, edema, and pneumonitis. To be safe, even mild hyperglycemia should be avoided or mitigated with metformin. Treatment is intended to be life-long, unless discontinued due to side effects.

Self-medication (even by physicians themselves) should be avoided and strongly discouraged. Instead, we need anti-aging clinics that implement the entire anti-aging recipe, including a complementary low carbohydrate diet and life style changes. Blood levels of rapamycin should be measured, as the rapamycin concentration in blood varies greatly among individuals taking the same dose. Doses of rapamycin should be tailored: personalized dosing and schedules. There is no shortage of potential patients who unfortunately already employ self-medication with rapamycin, but there is a shortage of physicians to treat them. Fortunately, a prototype clinic already functions in the USA, demonstrating that it is feasible from a regulatory standpoint (see Alan Green’s practice, Little Neck, NY). We cannot wait for results from others if we want to live longer and healthier ourselves. The time is now.

## Disclaimer

This article is addressed to clinical scientists and physicians. It is intended for informational and educational purposes only. Medical doctors interested in this topic may e-mail the author at **Blagosklonny@rapalogs.com**
